# Analytical study on cardiovascular implications of remote work, sedentary behavior and stress

**DOI:** 10.6026/973206300213059

**Published:** 2025-09-30

**Authors:** Biswarup Biswajit Chatterjee, Saranya Kuppusamy, Moe Thidar Aung, Ajeet Saoji, Sailesh I.S. Kumar

**Affiliations:** 1 Department of Emergency, Ruby General Hospital, West Bengal, India; 2 Department of Medical ICU, Sagar Hospitals, Jayanagar, Bangalore, Karnataka, India; 3 Department of General Medicine, Foundation Year 2 Doctor in General Medicine, Swansea Bay University Health Board, Wales United Kingdom; 4 Department of Community Medicine, NKP Salve Institute of Medical Sciences & Research Centre, Nagpur, Maharashtra, India; 5 Department of Surgery, Madras Medical College, Chennai, Tamilnadu, India

**Keywords:** Remote work, sedentary lifestyle, stress, cardiovascular health, lipid profile, blood pressure, cross-sectional study

## Abstract

Analysis of cardiovascular health markers among 142 adults engaged in remote work for a period 1 year is of interest. Participants were assessed for sedentary duration, perceived stress levels (using PSS-10) and cardiovascular parameters such as blood pressure, heart rate and lipid profile. Increased sedentary time (>8 hours/day) and higher stress scores were significantly associated with elevated systolic BP, LDL cholesterol and resting heart rate. Thus, we show the interlinked impact of prolonged remote work, inactivity and stress on cardiovascular risk.

## Background:

Over the past decade, the field of sedentary behavior (SB) research has expanded and evidence supporting a direct association between SB and cardiovascular disease (CVD) has emerged [1]. Moreover, the rise of remote work, accelerated by the global COVID-19 pandemic, has reshaped occupational structures worldwide. While it offers benefits such as flexibility and reduced commute time, remote work has also been linked to unintended health consequences-most notably, increased sedentary behavior and chronic stress [2]. Prolonged sitting reduced physical activity and lack of work-life boundaries can collectively heighten the risk for cardiovascular disease (CVD), which remains the leading cause of morbidity and mortality globally [3]. Sedentary behavior, defined as low-energy activities performed while sitting or reclining, is now recognized as an independent cardiovascular risk factor even among individuals who meet daily exercise recommendations [4]. Concurrently, the psychological toll of remote work characterized by isolation; blurred personal-professional boundaries and digital overload can activate chronic stress pathways that contribute to hypertension, dyslipidemia and other adverse metabolic changes [5]. Despite growing concern, data on the combined impact of remote work, sedentary behavior and stress on cardiovascular parameters remain limited, particularly in younger and middle-aged adults who form the bulk of the remote-working population [6]. Therefore, it is of interest to assess cardiovascular risk markers in remote workers and examine associations between sedentary time, perceived stress and cardiovascular health outcomes such as blood pressure, heart rate and lipid profile.

## Materials and Methods:

This analytical cross-sectional study was conducted between March and October 2024 in an urban tertiary wellness center. A total of 142 adult participants aged 25-55 years were recruited through corporate wellness programs and online platforms. Eligible participants were full-time remote workers for at least one year, with no prior diagnosis of cardiovascular disease. Exclusion criteria included pregnancy, known endocrine or cardiac disorders, recent acute illness, or regular antihypertensive or lipid-lowering therapy. Each participant completed a structured questionnaire including demographic details, daily working hours, average sedentary time, physical activity level (using the Global Physical Activity Questionnaire - GPAQ) and perceived stress using the validated Perceived Stress Scale (PSS-10). Participants were categorized into low (<13), moderate (14-26), or high (>27) stress groups. Clinical assessments included resting blood pressure (average of two seated readings), resting heart rate, body mass index (BMI) and waist circumference. Fasting blood samples were collected to evaluate lipid profile (total cholesterol, LDL, HDL, triglycerides) and fasting blood glucose. Sedentary time was classified into three groups: <6 hours/day, 6-8 hours/day and >8 hours/day. Data were analyzed using SPSS version 27. Descriptive statistics summarized baseline characteristics. ANOVA and Chi-square tests assessed associations between sedentary time, stress level and cardiovascular variables. Pearson correlation and multivariate regression were used to identify predictors of elevated BP and dyslipidemia. A p-value <0.05 was considered statistically significant.

## Results:

Out of 142 participants, the majority were in the 31-45 age group, with 59.2% being male. A high proportion (68.3%) reported sitting more than 8 hours daily. Elevated systolic blood pressure, LDL cholesterol and resting heart rate were significantly associated with longer sedentary time and higher perceived stress levels. Multivariate regression revealed that high stress and sedentary time were independent predictors of elevated cardiovascular risk markers. Table 1 (see PDF) shows the majority of participants were aged 31-45 and predominantly male. Table 2 (see PDF) shows most participants reported sitting for more than 8 hours daily. Table 3 (see PDF) shows Moderate to high stress levels were reported by 78.1% of participants. Table 4 (see PDF) shows with higher sedentary time had significantly elevated systolic BP. Table 5 (see PDF) shows Higher perceived stress was associated with increased resting heart rate. Table 6 (see PDF) shows Participants with higher sedentary time had lower HDL and higher LDL levels. Table 7 (see PDF) shows High stress levels correlated with elevated fasting blood glucose. Table 8 (see PDF) shows Participants reporting >8 hours of sedentary time had significantly higher BMI and waist circumference. Table 9 (see PDF) shows more than 40% of high-stress participants were hypertensive. Table 10 (see PDF) shows Multivariate regression identified stress and sedentary time as independent predictors of high systolic BP.

## Discussion:

This analytical cross-sectional study reveals a concerning relationship between remote work, prolonged sedentary behavior and perceived stress with key cardiovascular risk factors. The majority of participants were middle-aged adults working remotely for over a year, with a substantial proportion reporting daily sedentary time exceeding 8 hours. These individuals exhibited significantly elevated systolic blood pressure, resting heart rate, LDL cholesterol and waist circumference-core markers of cardiometabolic risk [7]. Sedentary time emerged as a powerful independent predictor of higher systolic blood pressure and unfavorable lipid profiles, even after adjusting for BMI and age. These findings support growing evidence that sedentary behavior exerts independent cardiovascular effects, likely through impaired glucose metabolism, increased sympathetic tone and systemic inflammation. The consistent association between low HDL, high LDL and prolonged sitting underscores the need for movement-integrated work routines [8]. Perceived stress levels, measured by the PSS-10, were also significantly correlated with adverse cardiovascular parameters, including fasting blood glucose and resting heart rate. Participants with high stress had nearly four times the odds of hypertension compared to low-stress counterparts, highlighting the strong link between psychological stress and sympathetic activation [9]. Moreover, over half of the high-stress group exhibited clustering of three or more cardiovascular risk factors, supporting the role of chronic stress as a catalyst for multisystem dysfunction. Interestingly, although age and gender were not independent predictors in regression models, BMI and stress levels significantly contributed to elevated systolic blood pressure [10]. This suggests that the remote work environment itself, through behavioral and emotional pathways, may be a modifiable driver of cardiometabolic burden-particularly in younger adults who may otherwise be perceived as low-risk. Our findings emphasize the need for structured wellness programs in remote work settings. Interventions should include regular breaks from sitting, virtual stress-reduction strategies, cardiovascular screening and employer-led initiatives promoting physical activity [11]. Public health policies must adapt to the remote work reality by targeting sedentary lifestyles and mental well-being as integral components of cardiovascular risk management.

## Conclusion:

Remote work is associated with prolonged sedentary time and increased stress, both of which are independently linked to adverse cardiovascular outcomes. Elevated blood pressure, dyslipidemia and clustering of multiple risk factors were significantly more prevalent among individuals with high sedentary exposure and stress levels. Incorporating preventive strategies focused on movement and mental health within remote work environments is essential to mitigate long-term cardiovascular risk.
